# Room for advancement: The regulatory fit of bottom‐rank intermediate feedback

**DOI:** 10.1002/ejsp.2374

**Published:** 2018-05-08

**Authors:** Melvyn R. W. Hamstra, Bert Schreurs

**Affiliations:** ^1^ Department of Organization and Strategy School of Business and Economics Maastricht University Maastricht The Netherlands; ^2^ Department of Business Economic and Social Sciences & Solvay Business School Vrije Universiteit Brussel Brussels Belgium; ^3^ School of Business and Economics Research Centre for Education and the Labour Market Maastricht University Maastricht The Netherlands

**Keywords:** performance ranking, regulatory focus, regulatory fit, engagement

## Abstract

This research tests the hypothesis that promotion‐focused individuals experience regulatory fit from bottom rank, intermediate performance‐feedback. Prior research suggests promotion‐focused individuals experience fit in high *social* ranks (power). Bottom *performance* ranks may appear psychologically opposite to high power, which might lead one to expect that promotion‐focused individuals experience fit at top ranks. We propose that the opposite occurs in intermediate performance ranking feedback, in that promotion‐focused individuals experience regulatory fit at a *bottom* rank, because bottom rank implies having something to gain (yielding eagerness), whereas top rank implies having something to lose (yielding vigilance). Study 1 (*N *=* *261) supports the notion that ranks affect eagerness/vigilance. Study 2 (*N *=* *199) extends these findings by examining engagement from regulatory fit.

Performance rankings, in which multiple individuals’ performance is ordered from low to high, are widely used in education, sports, and business, and have historically received attention in social psychology due to a longstanding interest in social comparison. By and large, performance rankings elicit a uni‐directional drive upward (Festinger, 1954), and high ranks are considered to hold higher value (Garcia, Tor, & Gonzalez, 2006); being the best often comes with bonuses or promotions, the status of a valedictorian speech, or the glory of a gold medal. Thus, being placed at a high rank is typically viewed as prompting stronger motivational effects (than other ranks), due to the value associated with a top rank (Vriend, Jordan, & Janssen, 2016). It is specifically those types of rankings that this research focuses on, which entail rewards for being the best.

Discussions about performance rankings, as the examples above illustrate, tend to revolve around individuals’ “final”—attained—rank. Yet, when individuals receive a rank as an intermediate performance indicator, their rank‐position may function as a reference point in ongoing performance. This notion has several interesting implications that have not been previously considered. In this research, we consider some of these implications from the perspective of regulatory focus theory (Higgins, 1997), which makes a distinction between promotion focus (eagerness to advance) and prevention focus (vigilance to maintain).

We propose that the type of intermediate rank feedback—bottom versus top rank—that individuals receive creates a frame of reference that affects their motivation for subsequent tasks (Scheepers, Ellemers, & Sassenberg, 2013). Specifically, when individuals receive intermediate feedback that their performance is *top* ranked, it implies they have attained something that they may subsequently lose again, and which they should be motivated to *maintain*; conversely, intermediate information that performance is *bottom* ranked implies there is something to gain, which individuals should be motivated to *advance* toward (Lount, Pettit, & Doyle, 2017; Scheepers et al., 2013). These two rank‐driven frames of reference are clear analogies to the frames that, according to regulatory focus theory (Higgins, 1997), prompt vigilance and eagerness, respectively.

Indirect support for this notion comes from Pettit, Yong, and Spataro (2010), who found the prospect of losing status led individuals to spend more time on subsequent tasks, suggesting vigilance. Moreover, Scheepers et al. (2013) demonstrated that decision‐makers in low (high) intergroup‐status groups tended to show eagerness (vigilance) in their decision‐making. Thus, top ranks imply non‐loss‐framing, which focuses individuals’ attention on maintenance and should, thus, yield vigilance (Hypothesis 1); bottom ranks imply gain‐framing, which focuses individuals’ attention on advancement and should, thus, yield eagerness (Hypothesis 2). This is not to say that situation‐specific ranking information would change people's regulatory focus. Rather, such effects are likely to be small and temporary and apply to the specific *strategic* state individuals experience toward the task at hand. According to regulatory focus theory, despite having strategic preferences, people adjust their goal‐pursuit strategy based on context, in this case the attained rank.

These hypotheses may be particularly interesting when considering their implications in the context of regulatory fit, a principle that predicts that individuals will feel engaged when they pursue goals in a way that sustains their motivational orientation (Freitas & Higgins, 2002; Higgins,^2000^). Examining the regulatory fit of *social* rank orderings, such as social power (Sassenberg, Ellemers, & Scheepers, 2012; Sassenberg, Jonas, Shah, & Brazy, 2007) and gender (Sassenberg, Brazy, Jonas, & Shah, 2013), studies find that promotion‐focused individuals experience regulatory fit in high power and high societal status positions due to the approach‐oriented, risky strategies that powerful positions (and the male gender‐stereotype) afford. A related theoretical domain to consider is that of performance feedback. Research suggests that promotion‐focused individuals tend to be motivated more by success feedback than by failure feedback (Förster, Grant, Idson, & Higgins, 2001), whereas the opposite pattern occurs in a prevention focus.

The studies on social rank‐orderings and on performance feedback might lead one to suspect that promotion‐focused individuals prefer or experience fit with *high* performance ranks. However, the regulatory fit hypothesis holds that engagement strength occurs from using *strategies* of goal‐pursuit that fit one's motivational orientation, independent of outcome value or success experience. That is, while promotion‐focused individuals could well value the rewards associated with having a top rank because they value the *outcome*, the value of the outcome is irrelevant for the occurrence of regulatory fit, as fit stems from the application of goal‐pursuit strategies that sustain the individual's regulatory focus. Perhaps somewhat counterintuitively, therefore, Hypotheses 1 and 2 suggest that promotion‐focused individuals should experience stronger engagement in a bottom‐rank position compared to a top‐rank (or no‐rank) position—that is, because the room for advancement encourages greater eagerness and lower vigilance (Hypothesis 3). Fit from a low rank, thus, represents a relatively unique context compared to prior fit research, as it implies both a low current standing and (intermediate) failure feedback. Accordingly, support for our hypothesis would provide a strong test of the regulatory fit hypothesis.

Although we have so far emphasized promotion focus, based on Hypotheses 1 and 2, one might argue that the effects of ranking are equally relevant in a prevention focus. We consider several reasons for why this may not be the case. First, performance rankings, as we conceptualize them, pertain to reward‐attainment, to which prevention‐focused individuals are not sensitive (Higgins et al., 2001). Second, because performance rankings create a uni‐directional drive upward (Vriend et al., 2016), performance rankings pertain primarily to the psychological domain of progress. Recent research (Zou, Scholer, & Higgins, 2014) indicates that the attainment of progress (gain) makes promotion‐focused individuals more motivated not to lose this gain, but does not affect prevention‐focused individuals the same way. Although the research on progress is comparable to our research on intermediate ranks, this research differs from Zou et al.'s (2014) research *and* from research on performance feedback (Förster et al., 2001) in examining (i) ranks as reference points that prompt eagerness and vigilance, and (ii) the regulatory *fit* hypothesis. We present two studies that use the same experimental paradigm, and in which people carry out a task twice, with ranking feedback in between the tasks. Hypotheses 1 and 2 are tested in Studies 1 and 2. Hypothesis 3 is tested in Study 2.

## Study 1

### Method

#### Participants

These were 261 individuals (59.0% female; *M*_age_ = 23.48, *SD*_age_ = 4.67), recruited from within thesis students’ networks at a business school to voluntarily participate in an online study (*N*_lowrank_ = 126, *N*_highrank_ = 135). Participants were mainly full‐time students (77.4%), some full‐time employed (15.7%), and the remaining 6.9% indicated another occupational status. Most participants indicated their native language as German (57.5%), Dutch (9.2%), or English (6.5%); 26.8% indicated another language. For Study 1, we assumed that 50 participants per cell would allow detecting the effect (considering an effect of 0.5 scale points and a *SD* of 1, a Cohen's *d* of 0.5). The fact that the sample size exceeds this is coincidental and not because of inspecting data during data collection.

#### Procedure

**Figure 1:**
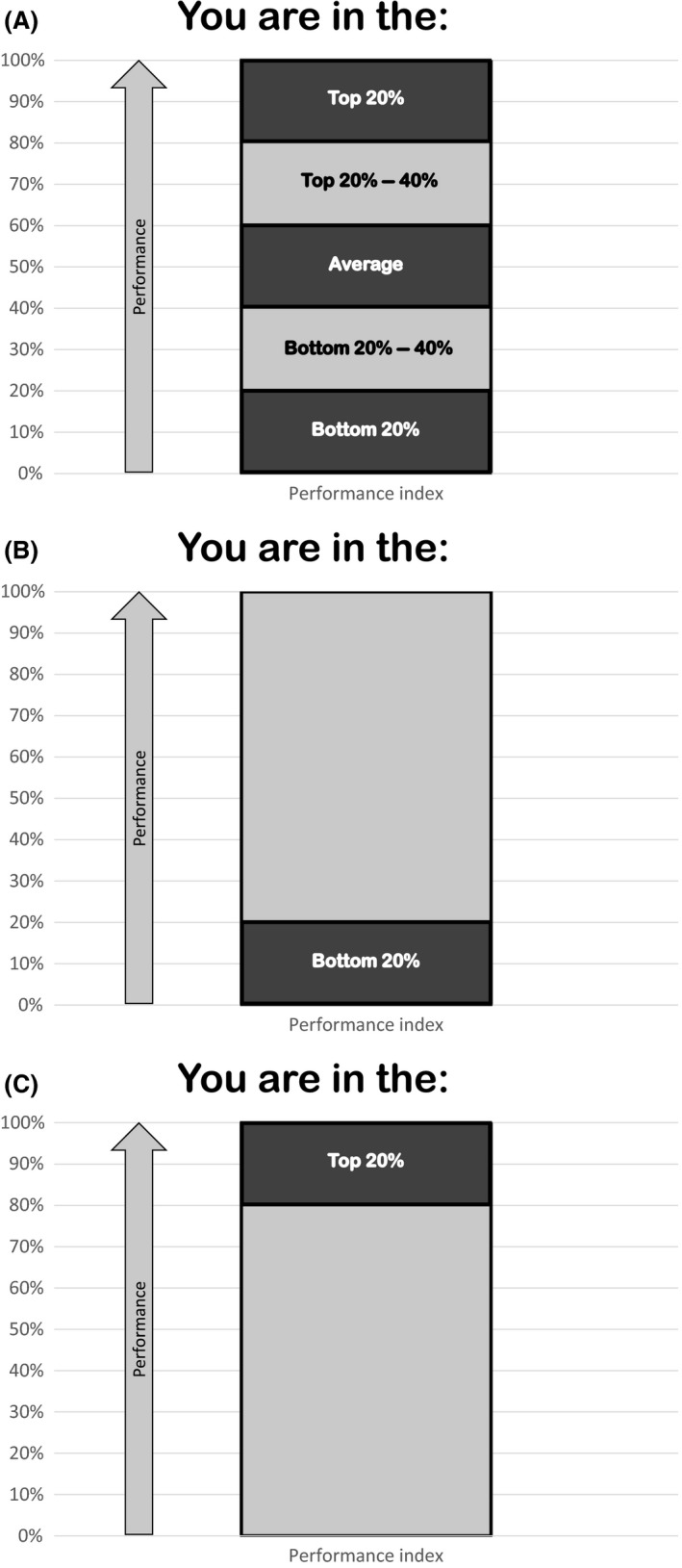
(A–C) Pictures used in the instruction and manipulation of the rank positions

Consistent with our theoretical assumptions about ranking, participants were informed that the three best‐performing participants would receive a gift certificate for €20. Participants were informed that they would receive information regarding their performance rank position after the task, and that they would then be allowed to try the task again (Figure 1A was used in the instructions). We refrained from providing an elaborate cover story or many explicit instructions (e.g., saying that the second task was meant to allow them to *improve* their performance) so as not to orient participants toward specific motivations. After Task 2, participants rated their eagerness and vigilance during the execution of the second task.

#### Measures and manipulations

##### Task description

Participants carried out a task in which they responded to 10 general knowledge statements that could be either true or false; they were given 20 seconds to respond to each. A correct answer on a statement meant a gain of 1 point; an incorrect answer meant a loss of 1 point. Participants could also click “no answer”, which meant neither gaining nor losing a point. The task was purposely designed to allow realistic variation in experienced eagerness (giving yes/no answers) and vigilance (giving no answers) and not to make the task particularly eager or vigilant. The statements were so difficult that no‐one would be able to know the answer by heart (e.g., The Democratic Republic of Congo has an approximate size of 2,344,800 km^2^). Statements were designed this way so that objective performance on the task would not become a possible confound.

##### Performance ranking

Top and bottom performance rank were manipulated with the pictures presented in Figure 1B,C, shown to participants after the first round of the task. We used the percentages as indicated in the pictures because we wanted these to be vague enough to be believable to all participants and because the proximity to top or bottom ranks creates similar psychological results as do specific ranks (Vriend et al., 2016). Moreover, we wanted to present participants with a simple visual presentation of the ordering to instill more strongly the sense of ordered performance from low to high.

##### Eagerness and vigilance

Participants indicated their eagerness (*M *=* *3.11, *SD *= 0.98; α = .82) and vigilance (*M *=* *3.47, *SD *= 0.94; α = .68) using three items each (e.g., “On the second task, I was eager to take all necessary actions” and “On the second task, I was vigilant and played it safe”).

### Results

Vigilance was higher in the high rank (*M *=* *3.57, *SD *= 0.93) compared with the low rank condition (*M *=* *3.35, *SD *= 0.93), *F*(1, 258) = 4.24, *p *=* *.041, η^2^_partial_ = .016. Eagerness was higher in the low rank (*M *=* *3.23, *SD *= 0.96) compared with the high rank condition (*M *=* *3.00, *SD *= 0.98), *F*(1, 258) = 4.05, *p *=* *.045, η^2^_partial_ = .015. Analyses controlled for the opposite strategy, which is the convention in regulatory focus research because it controls for the possibility that a more general effect on motivational strength explains an effect.

## Study 2

Study 1 supported the hypotheses that top ranks yield vigilance (Hypothesis 1) and bottom ranks yield eagerness (Hypothesis 2). Study 2 sought to examine the regulatory fit effect of low rank for promotion‐focused individuals (Hypothesis 3).

### Method

#### Participants

These were 199 individuals (61.3% female; *M*_age_ = 24.55, *SD*_age_ = 3.15) recruited in the same way as in Study 1 (but from other thesis students’ networks) to voluntarily participate in an online study (*N*_lowrank_ **= 66, *N*_highrank_ = 68, *N*_no‐rank_ = 65). Participants were mainly full‐time students (67.8%); some indicated being full‐time employed (27.1%) and the remaining 5.1% indicated another occupational status (one did not complete the question). Most of the participants indicated their native language to be German (77.9%), Dutch (9.0%), or English (3.5%); the remaining 9.0% indicated another language (one did not indicate language).

Based on the observed effects in Study 1, Study 2 might be underpowered for showing a main effect of rank on eagerness‐vigilance. However, the purpose of this study is to test the hypothesized fit effect. Although testing an interaction generally requires more power, this may be not the case for regulatory fit. Fit effects are stronger than main effects of regulatory focus or main effects of situational forces interacting with regulatory focus to create fit (Hamstra, Van Yperen, Wisse, & Sassenberg, 2014). Accordingly, we assumed a medium‐sized effect that could be detected with (again) 60 participants per cell.

#### Procedure

The procedure was identical to Study 1, with three exceptions. First, participants’ chronic regulatory focus was assessed before they received instructions. Second, a no‐rank comparison condition was included. Third, engagement strength was measured after Task 2.

#### Measures and manipulations

##### Chronic regulatory focus

We used Higgins et al.'s (2001) questionnaire to measure promotion focus (*M *=* *3.73, *SD* = 0.56; α = .60), and prevention focus (*M *=* *3.31, *SD* = 0.76; α = .72).

##### Task description

The task was the same as in Study 1, except that we allotted 30 seconds per trial instead of 20 seconds. Although Study 1 found support for the hypotheses, after reviewing the task, we considered the possibility that the time limit of 20 seconds might have created a too‐great sense of time pressure.

##### Performance ranking

The manipulation was the same as in Study 1. This time, we included a no‐rank condition. We explained, beforehand, that the study examined the effect of ranking information and that, for this reason, most, but not all participants would receive ranking information. We explained that participants would be randomly assigned to receive ranking information or not, and that not receiving ranking information would not suggest anything about actual performance. In the no‐rank condition, this last instruction was repeated after Task 1.

##### Eagerness and vigilance

After Task 2, participants were asked to indicate their level of eagerness (*M *=* *3.17, *SD *= 0.98; α = .84) and vigilance (*M *=* *3.05, *SD *= 1.04; α = .77) as in Study 1.

##### Engagement strength

We used a set of four bipolar items to which participants responded on five‐point scales, with the preamble “While I was doing the second task, I felt…” The response scales ranged from *disengaged* to *engaged,* from *bored* to *fascinated,* from *unimmersed* to *immersed* and from *uninvolved* to *involved* (*M *=* *3.03, *SD *= 1.01; α = .89).

### Results

#### Effect of rank on eagerness and vigilance

We constructed contrast tests that compare (i) eagerness in the low rank condition to the high rank and no‐rank condition combined, and (ii) vigilance in the high rank condition to the low rank and no‐rank condition combined. First, eagerness was higher in the low rank condition (*M *=* *3.34, *SD *= 0.89) compared with the high rank condition (*M *=* *3.13, *SD *= 1.05) and compared with the no‐rank condition (*M *=* *3.05, *SD *= 1.00). A contrast (just as in Study 1, controlling for the opposite strategy), as defined above, was supportive of the pattern but the *p* value was not below the .05 level, *C *=* *0.28, *SE* = 0.14, *p *=* *.051, Cohen's *d* = 0.286. Second, vigilance was higher in the high rank condition (*M *=* *3.21, *SD *= 1.03) compared with the low rank condition (*M *=* *2.97, *SD *= 0.99) and the no‐rank condition (*M *=* *2.96, *SD *= 1.09). A contrast was supportive of the direction, but was not statistically significant, *C *=* *0.26, *SE* = 0.15, *p *=* *.083, Cohen's *d* = 0.250. One may note that the contrasts used two‐sided tests.

#### Regulatory fit

To test the regulatory fit hypothesis, we conducted a moderated multiple regression analysis predicting engagement strength (see Table 1). Two dummy‐coded^1^ variables modeled the three conditions, with low rank as the reference category (both dummies are coded 0 for low rank; dummy 1 is coded 1 for no rank and 0 for high rank; dummy 2 is coded 1 for high rank and 0 for no rank). Predictors were chronic promotion focus, chronic prevention focus, the two dummy variables, and the interactions between the dummy variables and promotion focus and prevention focus. The non‐dichotomous predictors were standardized.

**Table 1:** Regression analysis results for Study 2 (*N* = 199; low rank is the reference category)

	Engagement				
	*B*	*SE*_*b*_	*t*	*p*	η^2^_partial_
Intercept	3.35	0.12	28.15	<.001	.81
Promotion focus	0.26	0.13	2.07	.040	.02
Prevention focus	−0.10	0.12	−0.83	.407	.00
Dummy 1 (no rank vs. low rank)	−0.55	0.17	−3.23	.001	.05
Dummy 2 (high rank vs. low rank)	−0.47	0.17	−2.80	.006	.04
Dummy 1 × Promotion focus	−0.52	0,17	−3.04	.003	.05
Dummy 2 × Promotion focus	−0.44	0.18	−2.50	.013	.03
Dummy 1 × Prevention focus	−0.03	0.17	−0.20	.844	.00
Dummy 2 × Prevention focus	0.15	0.17	0.84	.401	.00
*Model R‐Square*	0.11	*F*(8, 190) = 3.05, *p *=* *.003			
*Adjusted R‐square*	0.08				

Estimated engagement levels and 95% confidence intervals within different conditions at high and low promotion focus					
		Mean	Lower bound	Upper bound	
Low promotion focus	Low rank	3.09	2.74	3.44	
	High rank	3.06	2.72	3.40	
	No rank	3.06	2.75	3.37	
High promotion focus	Low rank	3.62	3.28	3.96	
	High rank	2.70	2.38	3.03	
	No rank	2.55	2.20	2.89	

**Figure 2:**
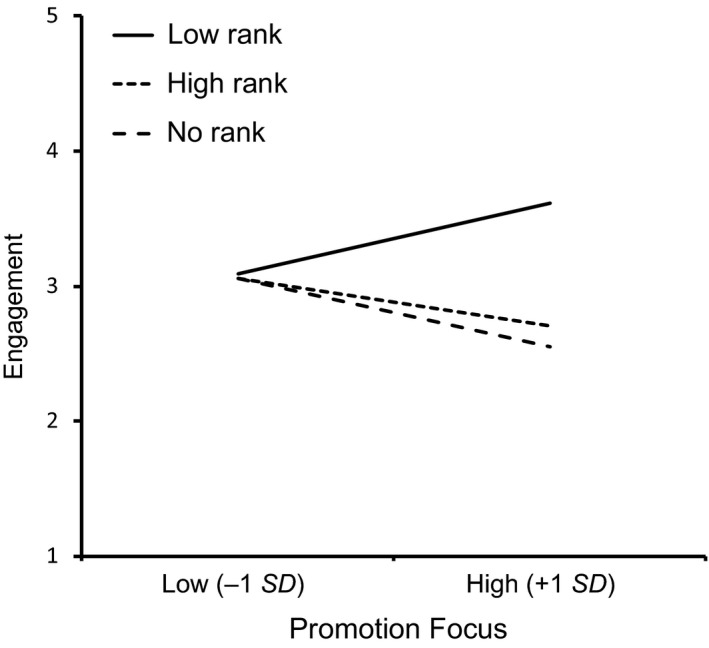
The interaction between performance rank conditions and promotion focus on sense of engagement

The interaction between promotion and the dummy contrasting low versus high rank, *B *=* *−0.44, *SE* = 0.18, *t*(190) = −2.50, *p *=* *.013, η^2^_partial_ = .032, and the interaction between promotion and the dummy variable contrasting low versus no rank, *B *=* *−0.52, *SE* = 0.17, *t*(190) = −3.04, *p *=* *.003, η^2^_partial_ = .046, were both significant. As Figure 2 depicts, highly promotion‐focused individuals felt more engaged in the low rank condition (*M *=* *3.62; 95% CI: 3.28; 3.96) than (i) in the high rank condition (*M *=* *2.70; 95% CI: 2.38; 3.03), *B *=* *−0.91, *SE* = 0.24, *t*(190) = −3.79, *p *<* *.001, η^2^_partial_ = .070, Cohen's *d* = 0.903, and (ii) in the no‐rank condition (*M *=* *2.55; 95% CI: 2.20; 2.89), *B *=* *−1.07, *SE* = 0.25, *t*(190) = −4.36, *p *<* *.001, η^2^_partial_ = .091, Cohen's *d* = 1.058. The difference between high rank and no‐rank conditions for highly promotion‐focused individuals was not significant, *B *=* *−0.16, *SE* = −0.65, *t*(190) = −0.65, *p *=* *.516, η^2^_partial_ = .002, Cohen's *d* = 0.155. At low promotion focus, there was no significant difference between any of the conditions, low (*M *=* *3.09; 95% CI: 2.74; 3.44) versus high rank (*M *=* *3.06; 95% CI: 2.72; 3.40), *B *=* *0.03, *SE* = 0.25, *t*(190) = 0.12, *p *=* *.904, η^2^_partial_ < .001, Cohen's *d* = 0.03, low versus no rank (*M *=* *3.06; 95% CI: 2.75; 3.37), *B *=* *0.03, *SE* = 0.24, *t*(190) = 0.13, *p *=* *.899, η^2^_partial_ < .001, Cohen's *d* = 0.03, high versus no rank, *B *<* *0.01, *SE* = 0.24, *t*(190) < 0.01, *p *=* *.999, η^2^_partial_ < .001, Cohen's *d* < 0.01.

Finally, none of the other interactions were significant: (i) the interaction between prevention and the dummy contrasting no versus high rank, *B *=* *0.18, *SE* = 0.17, *t*(190) = 1.05, *p *=* *.293, η^2^_partial_ = .006, (ii) the interaction between prevention and the dummy contrasting low versus no rank, *B *=* *0.03, *SE* = 0.17, *t*(190) = 0.20, *p *=* *.844, η^2^_partial_ < .001, (iii) the interaction between prevention and the dummy contrasting high versus low rank, *B *=* *0.15, *SE* = 0.17, *t*(190) = 0.84, *p *=* *.401, η^2^_partial_ = .004, and (iv) the interaction between promotion and the dummy contrasting high versus no rank, *B *=* *0.08, *SE* = 0.17, *t*(190) = 0.47, *p *=* *.639, η^2^_partial_ = .001. These results provide support for Hypothesis 3, that promotion‐focused individuals experience greater engagement in bottom‐rank position compared to a top‐rank (or no‐rank) position.

**Figure 3:**
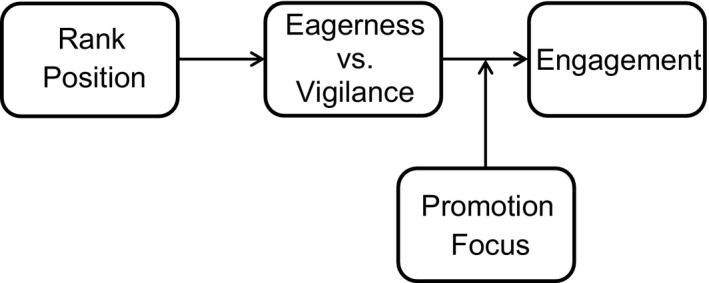
Graphical illustration of the indirect effect of rank on engagement via eagerness‐vigilance moderated by promotion focus

To analyze whether the observed regulatory fit effect may be attributed (as our argumentation implies, see Figure 3) to eager versus vigilant task‐experiences, we conducted a conditional indirect effects analysis using Hayes’ (2013) PROCESS macro. As mediator, we used the difference score eagerness minus vigilance to incorporate the relative nature of regulatory fit. The independent variable was a dummy that contrasts the focal condition (low rank) versus the two other conditions. The moderator is promotion focus and the analysis includes the same control variables as above (prevention focus and its interaction with the dummy). Thus, we tested whether the regulatory fit effect was mediated by eagerness versus vigilance at a high level, but not at a low level, of promotion focus. The analysis indeed indicated an indirect effect at high (+1 *SD*), *B *=* *0.07, *SEboot* = 0.05, 95% CI = 0.007; 0.216, but not at low (−1 *SD*) promotion focus, *B *=* *0.03, *SEboot* = 0.05, 95% CI = −0.038; 0.162.

## Discussion

Consistent with our hypotheses, vigilance was higher after top‐rank feedback (compared with bottom and no‐rank) and eagerness was higher after bottom‐rank feedback (compared with top and no‐rank). Although differences between conditions were nearly identical across the studies, in Study 2, differences were not significant at two‐sided 5% level. Most importantly, promotion‐focused individuals experienced greater engagement in performing the subsequent task after receiving intermediate bottom‐rank feedback, compared to both top‐rank and no‐rank feedback.

This research contributes to the regulatory fit literature. The regulatory fit hypothesis holds that engagement strength ensues from the use of fitting goal‐pursuit strategies, independent of value. However, it is often difficult to separate utility and strategic effects, making it difficult to test a pure strategic fit hypothesis. Top ranks are associated with greater value and in this research they were, too; yet, bottom rank elicited engagement among promotion‐focused individuals. Hence, results support the notion that strategies of goal‐pursuit affect engagement, even in a situation in which the more objective utility may appear lower.

The current research may also be interesting because regulatory focus theory is often interpreted as implying that success sustains promotion motivation (Förster et al., 2001). Yet, bottom‐rank feedback appears to constitute failure feedback. It seems that promotion‐focused individuals may not construe this as failure but may see it as “room for advancement”, eliciting eagerness. Hence, this research relates to the insights of Zou et al. (2014), that promotion‐focused individuals become more concerned with *losing* after experiencing gain. The current findings add to this by indicating that, in the context of performance ranking, promotion‐focused individuals become more engaged in *winning* after experiencing a non‐gain. There are important conceptual connections, such as the fact that in both Zou and colleagues’ work, and in the current research, feedback pertained to an uncompleted goal, operative being that individuals’ current position was not the final outcome.

One limitation of this research may be the relative disconnect between the ranking manipulation and the reward. The top‐rank feedback gave participants vague information that they were close to the top. Competition in proximity to top or bottom ranks already suffices to create the psychological effects of these reference points (Vriend et al., 2016). Nevertheless, it is plausible that the effect of ranking information differs according to the level of feedback‐specificity (e.g., explicitly stating that participants are now “in pole position to obtain the reward” versus “close to obtaining the reward”). This does not undermine the validity of our conclusions, because our purpose was to compare top and bottom relative to one another. Nevertheless, giving people concrete feedback that they are currently performing as one of the recipients of a reward reflects the possibility of losing an attained gain and should make people even more concerned about maintenance (Zou et al., 2014). A limitation that should be acknowledged is that Study 2 was relatively underpowered for testing the effect of ranking on eagerness‐vigilance.

Regulatory fit research does not typically use “neutral” conditions because no‐treatment comparison conditions generally do not yield a theoretically meaningful comparison. It is, accordingly, difficult to determine whether fit is engaging or misfit is disrupting. This research did include a control (no‐rank) condition. Finding that it resembles the “misfit” condition (top rank) suggests that regulatory fit, indeed, *adds* to engagement.

In the context of prior regulatory fit research on social ranks (e.g., power), the crucial difference between intermediately‐received rank‐feedback and social power and gender may be that performance ranks are relatively more changeable. This speculation is consistent with findings showing, for example, that lower‐power yields promotion‐oriented behaviors when power hierarchies are unstable (Sligte, De Dreu, & Nijstad, 2011). Therefore, future research may find that, in order to understand the effects of rank ordering, it is important to determine perceived stability of different orderings and, practically, to help low‐ranked individuals understand that there is room for advancement. Thus, a regulatory focus perspective on rankings adds to a basic understanding of how rank ordering works.

## Conflict of Interest

The authors confirm they have no conflict of interest to declare. Authors also confirm that this article adheres to ethical guidelines specified in the APA Code of Conduct as well as the authors' national ethics guidelines.

## Note

1 One could also (argue to) use orthogonal contrasts. Most logical would be a set in which contrast #1 codes low rank 2, and codes high and no rank −1, and contrast #2 would code low rank 0, no rank 1, and high rank −1. These two contrasts would test no rank and high rank together against low rank, and simultaneously would test whether for example engagement of promotion‐focused individuals does (not) differ between high vs. no rank). These contrasts would be more powerful but provide less information about differences between conditions.
